# Creation and Validation of the Brief Healthy Eating Habits Scale (BHEHS-6B, Version 1.0), Based on Harvard’s Healthy Eating Plate, in a Sample of Young, Middle-Aged, and Older Peruvian Adults

**DOI:** 10.3390/nu17111795

**Published:** 2025-05-26

**Authors:** David Javier-Aliaga, Gluder Quispe, José Anicama, Julio Mendigure Fernandez, Keila Miranda-Limachi, Yaquelin E. Calizaya-Milla, Norma Del Carmen Gálvez-Díaz, Luz Antonia Barreto-Espinoza, Jacksaint Saintila

**Affiliations:** 1 Faculty of Health Sciences, Universidad Peruana Unión, Lima 15464, Peru; 2 School of Psychology, Faculty of Psychology, Universidad Nacional Federico Villarreal, Lima 15088, Peru; 3 Research Group for Nutrition and Healthy Behaviors, School of Medicine, Universidad Señor de Sipán, Chiclayo 14001, Peru

**Keywords:** healthy eating habits, psychometric, factor analysis, young, middle-aged, older adults, Peru

## Abstract

**Background.** Healthy eating habits are essential for preventing chronic diseases and improving quality of life. However, there is a lack of brief and culturally adapted instruments for accurate assessment. Therefore, the aim of this study was to develop and validate the Brief Healthy Eating Habits Scale (BHEHS-6B, Version 1.0), based on Harvard’s Healthy Eating Plate, in a sample of young, middle-aged, and older Peruvian adults. **Methods.** The study followed a psychometric design. A non-probabilistic sample of 223 participants (both sexes; mean age = 41.6, SD = 15.8) was drawn from Metropolitan Lima, Peru. The BHEHS-6B (Version 1.0) was administered. **Results.** The bifactor model confirmed the unidimensional structural validity of the BHEHS-6B, showing acceptable global fit indices (CFI = 0.987, TLI = 0.937, SRMR = 0.025, RMSEA = 0.081) and an adequate hierarchical omega for the general factor (G = 0.638), supporting the use of a single total score. Finally, internal consistency was adequate for the total scale (α = 0.769, ω = 0.780). **Conclusions.** The BHEHS-6B is a valid and reliable instrument for assessing healthy eating habits, demonstrating evidence of strong content validity, internal consistency, and an adequate factor structure. Moreover, as a brief instrument, it is particularly useful for studies aiming to evaluate multiple variables and for the implementation of public health policies focused on improving community health.

## 1. Introduction

Eating habits play a fundamental role in the prevention of chronic non-communicable diseases (CNCDs), such as diabetes, hypertension, and obesity, which are among the leading causes of mortality worldwide [1]. According to the World Health Organization (WHO), NCDs account for 74% of deaths worldwide, many of which could be prevented through lifestyle changes—particularly by adopting healthy eating habits [2]. Similarly, in relation to overweight and obesity, if preventive measures are not implemented, global costs could reach three trillion USD per year by 2030 and exceed eighteen trillion USD by 2060 [3]. The assessment of eating habits has been a central focus in public health and nutrition research due to their direct influence on the development of NCDs. The early identification of inadequate dietary patterns is essential for implementing preventive strategies and promoting effective interventions within the population.

Various instruments have been developed to assess eating habits in different contexts. For example, one of the most widely used is the National Institutes of Health’s Eating Habits Questionnaire, which consists of 144 items and is designed to evaluate diet quality and consumption patterns [4]. Although this questionnaire has been widely validated, its length and complexity limit its applicability in contexts where quick and efficient assessment is required. For their part, Engelmann et al. [5] designed and validated the General Dietary Behavior Inventory (GDBI), a 16-item instrument based on the nutritional recommendations of the World Health Organization (WHO) and the German Nutrition Society (DGE). In Spain, Gila-Díaz et al. [6] designed and validated the Adherence to Healthy Food Pyramid Questionnaire (AP-Q), an instrument designed to evaluate adherence to the Healthy Food Pyramid (HFP) and which is a self-administered questionnaire consisting of 28 questions. Another relevant instrument is the Healthy Eating Index (HEI), developed by Eileen T. Kennedy et al. [7] at the United States Department of Agriculture (USDA) to assess the quality of the population’s diet in relation to the Dietary Guidelines for Americans. Although the HEI is a robust tool, its application requires detailed calculations and the collection of precise dietary intake data, which may be unfeasible in large-scale population studies.

Similarly, in the Latin American context, several research initiatives have focused on the development and validation of instruments to assess eating habits. For example, Pino et al. [8] developed and validated a 38-item questionnaire to measure eating behaviors and habits among primary health care users in Chile. Subsequently, the same tool was adapted and validated by Diaz et al. [9], with the items modified to suit a university context, resulting in a 35-item version. Similarly, in Mexico, Flores et al. [10] developed and validated a 26-item questionnaire. On the other hand, González et al. [11] created an 18-item self-assessment tool on eating habits for the adult population in Buenos Aires, Argentina. In Peru, the Eating Habits Questionnaire (EHQ), consisting of 32 items, was adapted and validated by Ferro and Maguiña [12]. The original 144-item instrument was developed in 2007 by the United States National Institutes of Health (NIH) [4]. The authors of the adaptation reported moderate reliability in the Peruvian context (α = 0.621). Similarly, other studies have also reported comparable levels of reliability [13,14,15].

Studies have employed a variety of tools with a considerable number of items to assess eating habits or healthy diets in young, middle-aged, or older adults. However, these instruments require more time to complete and are more susceptible to biases associated with participant fatigue or lack of interest [4,5,6,8,9,11,12,16,17,18,19,20,21,22,23,24,25,26,27,28]. However, there are few brief (fewer than 10 items) and validated instruments available for assessing this construct [29,30,31]. In this sense, there is a knowledge gap regarding the availability of brief, validated and culturally adapted scales for young, middle-aged, and older adults. On the other hand, although the aforementioned instruments have proven useful for assessing eating habits in various contexts, their use may pose a barrier in studies that require a more agile and less demanding application for participants. The length of such instruments can limit their practical applicability in contexts where brief, accessible, and reliable tools are needed—especially in research involving large samples and multiple variables that require the use of complex statistical models [29,30,31]. In this context, and with the aim of providing a brief, accessible, and effective alternative for assessing healthy eating habits, the Brief Healthy Eating Habits Scale (BHEHS-6B, Version 1.0) was developed, consisting of six items.

One of the most influential nutritional guidelines today is Harvard’s Healthy Eating Plate, developed by nutrition experts at the Harvard T.H. Chan School of Public Health in collaboration with the editors of Harvard Health Publications. Due to its clarity, practicality, and strong scientific foundation, this model has become an international benchmark in the promotion of healthy eating. This model presents a visually balanced distribution of the main food groups required for a healthy diet: half of the plate should consist of fruits and vegetables (with an emphasis on vegetables), one quarter of whole grains, and the remaining quarter of healthy protein sources. It also encourages the use of healthy vegetable oils instead of trans or saturated fats and recommends water as the primary beverage. In summary, a healthy diet should include vegetables, fruits, whole grains, healthy proteins, vegetable oils, and water [32]. In this context, the BHEHS-6B was developed based on the Healthy Eating Plate proposed by Harvard, as well as recent advances in nutritional science. Notably, to date, no instrument has been identified that specifically assesses healthy eating habits based on Harvard’s Healthy Eating Plate.

The BHEHS-6B has the following main features: it is a brief instrument designed to minimize respondent fatigue and associated biases. Additionally, it was developed for use in research involving large samples, multiple variables, or public health programs focused on the prevention and promotion of healthy eating habits. The BHEHS-6B provides a general assessment of healthy eating habits, offering a comprehensive and practical overview of dietary behavior. Unlike instruments that evaluate diet in detail—such as 24-h dietary recalls—this scale does not focus on the specific quantification of nutrients, but rather on eating patterns and behaviors associated with a healthy diet. In other words, while traditional tools measure the nutritional content of consumed foods, the BHEHS-6B emphasizes the habits that reflect a balanced and health-promoting diet.

In this context, the present study aims to develop and validate the BHEHS-6B, based on Harvard’s Healthy Eating Plate, in a sample of young, middle-aged, and older Peruvian adults. The general hypothesis proposed is that the BHEHS-6B is a valid and reliable instrument for the global assessment of healthy eating habits in this population. Specifically, the following hypotheses are proposed: (1) (H_1_) The BHEHS-6B demonstrates adequate content validity, as evaluated by expert judges. (2) (H_2_) The BHEHS-6B shows adequate internal consistency, with acceptable Cronbach’s alpha and omega values. (3) (H_3_) The BHEHS-6B presents a valid general structure, as evidenced by a bifactor model. (4) (H_4_) The structure of the BHEHS-6B can also be represented through two dimensions: (1) healthy eating habits related to micronutrients and water (dimension 1), and (2) habits related to macronutrients (dimension 2), as evidenced by confirmatory factor analysis. 

## 2. Materials and Method

### 2.1. Study Design and Participants

The study employed a cross-sectional and psychometric design, as it focused on the development and validation of the BHEHS-6B [33]. The sample consisted of 223 participants from Metropolitan Lima, Peru, selected through non-probability snowball sampling [34]. The inclusion criteria were as follows: Peruvian nationals of both sexes, aged between 18 and 79 years, who provided informed consent. Conversely, the exclusion criteria included participants who completed the surveys only partially. Data collection was conducted in January 2025.

Table 1 shows that the participants had an average age of 42.29 years (SD = 15.48), with an age range from 18 to 79 years. Regarding gender, the majority were women (54.3%), while men represented 45.7% of the sample. In terms of educational level, most participants had a university education (60.5%), approximately one-quarter had technical training (22.0%), and a minority had only a secondary education (17.5%). With regard to marital status, nearly half of the sample were single (48.3%), followed by married participants (43.4%), while divorced and widowed individuals represented a small proportion (5.5% and 2.8%, respectively). Finally, in terms of employment status, more than half of the participants were self-employed (53.9%), while the remainder (46.1%) held dependent employment.

### 2.2. Instruments

#### Brief Healthy Eating Habits Scale (BHEHS-6B, Version 1.0)

The BHEHS-6B (Version 1.0) is composed of six items. This instrument can be interpreted using either a unidimensional or a bidimensional structure. Dimension 1, titled “healthy eating habits related to micronutrients (vegetables, fruits) and water,” includes items 1, 2, and 3. Dimension 2, titled “healthy eating habits related to macronutrients (grains, tubers, proteins, and vegetable fats),” comprises items 4, 5, and 6. Responses are recorded on a five-point Likert scale ranging from “never” (1) to “always” (5). The total score is calculated by summing the responses to all items, yielding a range from 6 to 30 points, with higher scores indicating healthier eating habits. The psychometric results of the BHEHS-6B are as follows: Content validity was confirmed through Aiken’s V values ranging from 0.93 to 1.00 (95% CI [0.62, 1.00], *p* < 0.05). The bifactor model supported the unidimensional structural validity of the BHEHS-6B, showing adequate global fit indices (CFI = 0.987, TLI = 0.937, SRMR = 0.025, and RMSEA = 0.081), as well as an acceptable hierarchical omega for the general factor (G = 0.638), which justifies the use of a total score. In turn, the confirmatory factor analysis supported a two-dimensional structure, also showing good model fit (CFI = 0.981, TLI = 0.965, SRMR = 0.0354, and RMSEA = 0.0601). Internal consistency was adequate for the total scale (α = 0.769, ω = 0.780), as well as for the first dimension (α = 0.712, ω = 0.724), and for the second dimension (α = 0.703, ω = 0.740). The BHEHS-6B is intended to provide a general assessment of healthy eating habits, primarily for use in future research and public health programs. The scale allows for a global evaluation of the construct or, alternatively, for separate analysis of its dimensions: healthy eating habits related to micronutrients (dimension 1) or those related to macronutrients (dimension 2). Healthy eating habits are defined as a set of behaviors associated with the consumption of foods that provide essential micronutrients and macronutrients. In this framework, habits related to micronutrients refer to behaviors aimed at the regular consumption of foods rich in vitamins and minerals, such as vegetables, fruits, and water. On the other hand, habits related to macronutrients involve behaviors linked to the adequate intake of foods that supply carbohydrates (such as grains and tubers), proteins, and healthy fats of plant origin [32,35,36,37,38,39,40,41,42,43]. The use of the BHEHS-6B is recommended by employing raw scores to assess both the total score and each of its two underlying dimensions. Additionally, a preliminary diagnostic classification based on raw scores is proposed—subject to future validation—which includes five levels: very low (6–12), low (13–18), moderate (19–23), high (24–27), and very high (28–30). The BHEHS-6B (Version 1.0) is the intellectual property of the primary author of this study (© 2025, David Javier-Aliaga [D.J.-A.]. All rights reserved). Any use of this scale in scientific publications, research projects, or other forms of dissemination requires prior written authorization (davidjavieraliaga@gmail.com). D.J.-A. is the legal rights holder and the beneficiary of the corresponding royalties. The full instrument is available in Appendix A (English version) and in Appendix B (Spanish version).

### 2.3. Theoretical Foundation of the BHEHS-6B

As mentioned in the introduction, the BHEHS-6B was developed based on Harvard’s nutritional model (Harvard’s Healthy Eating Plate) and the current scientific knowledge of nutrition. Table 2 presents the theoretical foundation of the items comprising the BHEHS-6B, showing the direct correspondence between the components of Harvard’s model and the items included in the scale [32]. Table 3 outlines the theoretical foundation of the two dimensions that structure the BHEHS-6B, which are defined according to the nutritional knowledge that distinguishes between micronutrients and water on the one hand, and macronutrients on the other [35,36,37,38,39,40,41].

### 2.4. Statistical Analysis

For data processing and analysis, the information collected was tabulated using SPSS version 29 and R Studio version 4.1.2. Descriptive analyses included frequency tables, standard deviation, skewness, and kurtosis. Psychometric analyses were conducted using Aiken’s V, Cronbach’s alpha (α), McDonald’s omega (ω), polychoric correlation, Pearson’s correlation, the bifactor model, hierarchical omega (ωh), and confirmatory factor analysis (CFA). Additionally, to enhance the clarity, grammar, and coherence of the manuscript, the authors used the language model ChatGPT (OpenAI, version GPT-4) in a supervised manner as an auxiliary tool for English editing and stylistic refinement. A significance level of 5% and a 95% confidence interval were considered throughout the study.

## 3. Results

### 3.1. Content Validation Analysis

Table 4 presents the results of the content validation analysis of the BHEHS-6B, based on evaluations provided by five expert judges, including two specialists in nutrition, two in public health, and one in health psychology. The scale was assessed using three key criteria: relevance, representativeness, and clarity. The average score (M) for each item and criterion was consistently high—generally 3.00—with a standard deviation (SD) of 0.00 in most cases, indicating full agreement among the expert judges. For items BHEHS1, BHEHS2, BHEHS3, and BHEHS4 across all three criteria, the Aiken’s V value was 1.00, interpreted as valid, with confidence intervals (CIs) ranging from 0.70 to 1.00, confirming the strong content validity of these items. In contrast, for items BHEHS5 and BHEHS6, some criteria—such as the representativeness of BHEHS5 and the relevance and clarity of BHEHS6—showed slightly lower average ratings of 2.80, with a standard deviation of 0.45 and an Aiken’s V value of 0.93, accompanied by confidence intervals ranging from 0.62 to 0.99. These results are still interpreted as valid, although they indicate slight disagreement among the judges. In summary, the Aiken’s V values and confidence intervals reflect high content validity for all six items of the BHEHS-6B, suggesting that the judges considered the items to be relevant, representative, and clear for assessing healthy eating habits.

### 3.2. Descriptive Statistics and Reliability

Table 5 presents the descriptive statistics and reliability analysis of the BHEHS-6B. The total score on the BHEHS-6B had a mean of 22.686 and a standard deviation of 3.283, with an asymmetry (A) of 0.279 and a kurtosis (K) of −0.531. Both values fall within the acceptable ±1.5 range, indicating a distribution that is approximately normal, with a slight positive skew and a somewhat flattened shape. Overall reliability was adequate, with a Cronbach’s alpha (α) of 0.769 and a McDonald’s omega (ω) of 0.780, indicating good internal consistency for the scale as a whole. Regarding the dimensions of the BHEHS-6B, dimension 1 had a mean of 10.807 (SD = 1.927), with an asymmetry (A) of 0.151 and a kurtosis (K) of −0.561. Both values fall within the ±1.5 range, indicating a normal distribution. The reliability coefficients for this dimension were also adequate, with α = 0.712 and ω = 0.724, indicating acceptable internal consistency. Dimension 2 had a mean of 11.879 (SD = 1.903), with an asymmetry (A) of 0.005 and a kurtosis (K) of −0.716—both within the ±1.5 range—reflecting a symmetrical and slightly flattened distribution. The reliability values for this dimension were α = 0.703 and ω = 0.740, also indicating good internal consistency. In conclusion, all skewness and kurtosis values for the BHEHS-6B were within the acceptable range, indicating that the data distributions were approximately normal. Furthermore, the BHEHS-6B demonstrated adequate reliability for both the total score and its individual dimensions, with alpha and omega coefficients reflecting good internal consistency. These findings support its use as a reliable instrument for assessing healthy eating habits in the target population.

Table 6 presents the descriptive statistics and reliability analysis for the items of the BHEHS-6B. In descriptive terms, item means (M) range from 3.56 to 4.17, with standard deviations (SD) ranging from 0.690 to 0.982, indicating a moderate level of response dispersion. Skewness (A) and kurtosis (K) values for each item fell within the ±1.5 range, suggesting distributions close to normality across all items. Regarding reliability, McDonald’s omega (ω) for each item, when removed from the full scale, ranged from 0.717 to 0.753, indicating that no item significantly reduced the overall internal consistency of the scale. The corrected item-total correlations (*ritc*), which range from 0.468 to 0.598, indicate adequate values, suggesting that all items contribute positively to the overall scale. The polychoric correlations between items were generally moderate, ranging from 0.288 to 0.652, reflecting that the items are independent yet related—an ideal characteristic for a brief scale. In particular, item BHEHS4 showed a relatively high polychoric correlation with BHEHS5 (0.725), indicating that these items share a stronger relationship compared with other item pairs, possibly reflecting related aspects within the construct of healthy eating habits. In summary, the BHEHS-6B items demonstrated solid psychometric properties, including symmetrical distributions, adequate corrected item-total correlations, and omega values that support the internal consistency of the scale.

### 3.3. Bifactor Analysis and Hierarchical Omega

Table 7 presents the structural validity of the instrument from a unidimensional perspective using the bifactor model. The results indicate adequate global model fit (CFI = 0.987, TLI = 0.937, SRMR = 0.025, RMSEA = 0.081). Specifically, the CFI and SRMR values reflect excellent model fit, while the TLI falls within an acceptable range, close to the ideal threshold (≥0.95). Although the RMSEA value was slightly above the conventional cutoff (<0.08), its magnitude is still interpretable as indicating a reasonable fit. Likewise, the hierarchical omega (ωh) for the general factor (G = 0.638) was interpreted as moderate to high, supporting the use of a global total score for the BHEHS-6B. This coefficient indicates that 63.8% of the explained variance in the instrument can be attributed to the general factor, which is considered an acceptable level of reliability. Overall, these results support the presence of a general factor, suggesting that the BHEHS-6B can be used through its total score.

### 3.4. Confirmatory Factor Analysis

To additionally evaluate the bidimensional structure of the BHEHS-6B, a confirmatory factor analysis (CFA) was conducted. Table 8 presents the results of the confirmatory factor analysis (CFA) for the Brief Scale of Healthy Eating Habits (BHEHS-6B), including the factor loadings of each item on the two identified factors. For the first factor, items BHEHS1, BHEHS2, and BHEHS3 showed unstandardized factor loadings of 0.592, 0.588, and 0.449, respectively—all statistically significant, with Z-values greater than 7 and *p* < 0.001. The 95% confidence intervals for these items fall within appropriate ranges, confirming a significant association with the first factor. Standardized factor loadings range from 0.535 (BHEHS3) to 0.778 (BHEHS1), suggesting a moderate to strong relationship between the items and the first factor. This indicates that the items effectively represent this dimension of healthy eating habits. Regarding the second factor, items BHEHS4, BHEHS5, and BHEHS6 showed unstandardized factor loadings of 0.564, 0.498, and 0.532, respectively, all statistically significant with *p* < 0.001 and high Z-values. The 95% confidence intervals for these items also indicate consistent associations with the second factor. The standardized factor loadings were moderate to high, with BHEHS4 showing the highest loading (0.820), indicating a strong and direct association with the second factor. In conclusion, the proposed bidimensional structure is confirmed, as the results of the confirmatory factor analysis support the existence of two dimensions in the BHEHS-6B.

Table 9 presents the results of the confirmatory factor analysis regarding the covariance between the two factors of the BHEHS-6B. The covariance between dimension 1 and dimension 2 was 0.622, with a standard error (SE) of 0.0646. The 95% confidence interval (CI) for this estimate ranged from 0.495 to 0.748, indicating a statistically significant relationship between the two dimensions. The Z-value of 9.63 with *p* < 0.001 reinforces the significance of this covariance, suggesting a positive and moderate association between the two dimensions. The standardized estimate was also 0.622, confirming a moderate correlation. This finding implies that, although each dimension captures distinct aspects of healthy eating habits, they are significantly related—indicating that both contribute jointly to the overall construct measured by the BHEHS-6B.

Table 10 presents the model fit indices from the confirmatory factor analysis of the BHEHS-6B. The results indicate a good model fit, with a comparative fit index (CFI) of 0.981 and a Tucker–Lewis index (TLI) of 0.965—both exceeding the 0.95 threshold—suggesting excellent fit to the data. The standardized root mean square residual (SRMR) was 0.0354, and the root mean square error of approximation (RMSEA) was 0.0601—both below the recommended cutoff of 0.08—indicating adequate model fit. The 90% confidence interval for the RMSEA ranged from 0.000 to 0.109, which includes values close to zero, suggesting acceptable fit overall, although the upper bound indicates that the model may not provide optimal fit in all cases.

Finally, the exact fit test (χ^2^ = 14.4, df = 8, *p* = 0.071) was not significant, indicating no substantial difference between the theoretical model and the observed data, thus supporting the adequacy of the model. Taken together, these indicators suggest that the proposed factor model for the BHEHS-6B fits the data well, confirming that the model structure is appropriate for measuring healthy eating habits in the studied sample.

## 4. Discussion

Eating habits play a key role in the prevention of CNCDs, such as diabetes, hypertension, and obesity, which account for 74% of deaths worldwide [1,2]. Without preventive measures, the global costs of overweight and obesity could exceed 18 trillion USD by 2060 [3]. Given its impact on public health, evaluating eating habits enables the identification of inadequate patterns and the development of preventive public health strategies. However, despite advances in the assessment of eating habits, there is still a lack of brief, validated, and culturally adapted scales for young, middle-aged and older adults, particularly within the Peruvian context. In particular, no instrument has been identified that specifically evaluates healthy eating habits based on the Harvard Healthy Eating Plate, which promotes a balanced diet including fruits, vegetables, whole grains, healthy proteins, healthy oils, and water [32]. Therefore, the aim of this study was to develop and validate the Brief Healthy Eating Habits Scale (BHEHS-6B, Version 1.0), based on the Harvard Healthy Eating Plate, for use in the Peruvian population. The discussion is presented below in terms of content validity, internal consistency, and construct validity (both unidimensional and bidimensional).

Regarding the content validity of the BHEHS-6B (Version 1.0), two key aspects were considered. First, the instrument was developed based on Harvard’s Healthy Eating Plate, through a structured adaptation of this nutritional model into a healthy eating habits scale format [32]. In other words, the BHEHS-6B is grounded in a pre-established theoretical model, which justifies the decision to omit an exploratory factor analysis (EFA) and instead conduct a confirmatory factor analysis (CFA) to assess its alignment with the proposed structure. Second, an expert judgment procedure was carried out to evaluate the adequacy, relevance, and representativeness of the items in relation to Harvard’s nutritional model and the scientific literature [35,36,37,38,39,40,41]. The results of the content validation showed that the five expert judges evaluated the BHEHS-6B items as relevant, representative, and clear. This indicates that the items of this new instrument adequately reflect healthy eating habits according to the established theoretical framework. The results show mean scores of 3.00 and Aiken’s V values ranging from 0.93 to 1.00, confirming the content validity of the instrument. Additionally, the confidence intervals (CIs) ranged from 0.62 to 1.00, with *p*-values < 0.05 in all cases, reinforcing the robustness of the expert evaluation. These findings indicate a high level of agreement among the judges, supporting the appropriateness of the selected items for measuring healthy eating habits. The absence of low scores in the expert evaluations suggests that the scale is understandable and appropriate for the target population, minimizing ambiguities or potential misinterpretations during its application. The content validity of the BHEHS-6B is consistent with previous studies that have developed similar instruments to assess eating habits. For example, the scale developed by Gabe et al. [20], based on the Brazilian Food Guide, employed similar approaches to validate content, yielding comparable results in terms of expert consensus. However, unlike these more extensive instruments, the BHEHS-6B is distinguished by its brevity, which facilitates its use in population-based studies without compromising psychometric rigor. Likewise, the empirical support obtained through the content validation of the BHEHS-6B aligns with established criteria for developing valid and culturally relevant psychometric instruments.

In terms of internal consistency, the overall reliability of the BHEHS-6B was supported by a Cronbach’s alpha (α) of 0.769 and a McDonald’s omega (ω) of 0.780, indicating good internal consistency for the scale as a whole. With regard to the dimensions, dimension 1 showed reliability coefficients of α = 0.712 and ω = 0.724, while dimension 2 presented values of α = 0.703 and ω = 0.740, suggesting that both dimensions demonstrate acceptable internal consistency. These results are consistent with findings reported for various instruments developed in different contexts. For example, the Healthy Eating Practices Scale by Gabe et al. [20], the Healthy Diet and Lifestyle Scale (DEVS) by Calisaya-Milla et al. [27], and the Acquired Healthy Lifestyle Assessment Scale (E-VEVSA) by Rodríguez et al. [21] have all demonstrated adequate levels of internal reliability. Moreover, it is important to highlight that the BHEHS-6B demonstrates higher internal consistency when compared with one of the most widely used questionnaires in the Peruvian context: the Eating Habits Questionnaire (EHQ), validated by Ferro and Maguiña, which reported a Cronbach’s alpha coefficient of 0.621 [12]. In this regard, the results of the present study support the conclusion that the BHEHS-6B possesses solid psychometric properties in terms of internal consistency, both overall and within its two specific dimensions. This reinforces the utility of the instrument as a brief yet reliable measure for assessing healthy eating habits. The stability of scores across items and dimensions suggests that the BHEHS-6B items consistently capture the eating behaviors they are intended to measure.

Regarding construct validity, the bifactor model showed an adequate overall fit (CFI = 0.987, TLI = 0.937, SRMR = 0.025, RMSEA = 0.081, χ^2^ = 7.346, *p* = 0.062), along with an acceptable hierarchical omega value for the general factor (G = 0.638). These results support the use of the BHEHS-6B through a global total score. On the other hand, the confirmatory factor analysis supported a two-dimensional structure, with standardized factor loadings ranging from 0.535 to 0.820 and good model fit indices (CFI = 0.981, TLI = 0.965, SRMR = 0.0354, RMSEA = 0.0601). These results confirm the hypothesis grounded in nutritional science, which proposed a two-dimensional structure for the BHEHS-6B. In other words, the observed data fit the bidimensional model of the instrument, supporting its structural validity. This finding is consistent with previous studies that have also reported confirmatory factor analyses, such as those conducted by Gabe et al. [20] and Calisaya-Milla et al. [27]. Similarly, when compared with other brief instruments—such as those proposed by Caldwell et al. [31], Lafrenière et al. [30] and Lara-Breitinger et al. [29]—the BHEHS-6B stands out as the only scale that provides formal statistical evidence of structural validity through confirmatory factor analysis. This indicates that, unlike other brief instruments whose validity relies mainly on external correlations or predictive models, the BHEHS-6B has empirically demonstrated that its items are also organized into two clearly defined and statistically supported dimensions. Therefore, the CFA findings for the BHEHS-6B consolidate its usefulness as a psychometric instrument with adequate construct validity, supporting its application in the brief assessment of healthy eating habits. Finally, it is worth noting that, as demonstrated in the study by Lafrenière et al. [30] using the Brief Diet Quality Assessment Tool, short instruments can be equally effective in assessing eating habits—provided they undergo rigorous psychometric validation. In this regard, the BHEHS-6B stands out as a flexible and reliable tool for evaluating adherence to a healthy diet. 

### 4.1. Implications for Public Health and Community Health

The implementation of the BHEHS-6B in public health studies or programs can significantly contribute to identifying inadequate dietary patterns within the population and to developing early intervention strategies aimed at preventing non-communicable diseases (NCDs). The availability of a brief, validated instrument to assess healthy eating habits is crucial for informing the design of effective health promotion policies and programs. At the community level, the BHEHS-6B can be used by health professionals in primary care settings, as well as by educators in educational, religious, workplace, and broader social contexts, to monitor and promote changes in healthy eating habits.

### 4.2. Psychometric Implications for Future Studies

Due to its brevity and sound psychometric properties, the BHEHS-6B represents a valuable tool for research involving multiple variables and the development of complex statistical models. In studies requiring advanced statistical analyses—such as multiple regression, structural equation modeling, or neural networks—it is essential to employ brief and efficient instruments that minimize participant burden without compromising measurement quality. Furthermore, the BHEHS-6B is well suited for use in large-scale cross-sectional studies, as well as in longitudinal, epidemiological, and interdisciplinary research aimed at examining the relationship between eating habits and various physical, psychological, or spiritual health outcomes. Finally, it may also be applied in studies involving manipulative strategies—such as quasi-experimental, and single-case designs—to evaluate the effects of nutritional or public health interventions. This would allow for the assessment of their effectiveness in monitoring adherence to healthy diets and measuring changes in eating habits over time [33].

### 4.3. Limitations and Future Studies

Despite the strong psychometric findings of the BHEHS-6B, this study has some limitations that should be acknowledged. First, the sample was selected through non-probabilistic sampling, which may limit the generalizability of the results to other populations or contexts. Future research should aim to expand the sample and include participants from different regions of Peru or other countries to ensure the representativeness and cross-cultural validity of the scale in diverse sociocultural and economic contexts.

Second, future research should explore its predictive validity in relation to metabolic health indicators such as body mass index (BMI), lipid profile, and others.

Third, although the BHEHS-6B was designed as a general instrument for assessing healthy eating habits, this feature also represents a limitation. Its broad focus prevents a detailed analysis of specific eating behaviors and restricts its applicability in clinical assessment contexts, where a more in-depth and individualized analysis of dietary patterns is required.

Fourth, an additional limitation to consider is that the BHEHS-6B assesses only healthy eating habits, without taking into account those that are unhealthy.

Finally, it is recommended that future studies validate the instrument using alternative statistical methodologies, such as neural network analysis or Bayesian factor analysis. It would also be pertinent to evaluate factor invariance across different groups and cultural contexts to ensure the structural stability of the scale in diverse populations.

## 5. Conclusions

The Brief Healthy Eating Habits Scale (BHEHS-6B, Version 1.0), developed based on the recommendations of the Harvard Healthy Eating Plate, is a valid and reliable instrument for assessing healthy eating habits among Peruvian youth, middle-aged, and older adults with characteristics similar to those of this study. Its psychometric properties are supported by analyses of content validity, internal consistency, and construct validity. The application of the BHEHS-6B is particularly valuable in studies involving multiple variables, as its brevity and solid psychometric properties allow it to be used without increasing respondent burden—an especially useful feature in research employing complex statistical models. Likewise, its use in epidemiological studies and large-scale research can provide valuable insights for the formulation of public policies aimed at improving eating habits and preventing diet-related diseases. In conclusion, the BHEHS-6B represents a significant contribution to the assessment of eating habits in academic and community settings, supporting the advancement of public health strategies aimed at improving population quality of life.

## Figures and Tables

**Table 1:** Sociodemographic data of the study sample.

		n/M	%/SD
**Age**		M = 42.29	SD = 15.48
**Sex**	Male	102	45.7
	Female	121	54.3
**Level of education**	Secondary	39	17.5
	Technical	49	22.0
	University	135	60.5
**Relationship status**	Married	93	43.4
	Single	107	48.3
	Divorced	15	5.5
	Widowed	8	2.8
**Employment status**	Employee	103	46.1
	Self-employed	120	53.9

**Note.** M = Average: SD = Standard deviation.

**Table 2:** Theoretical foundation of the items and the unidimensional structure of the BHEHS-6B.

Nº	Components of Harvard’s Healthy Eating Plate	Items of the BHEHS-6B
1	Vegetables	BHEHS1: Do you consume vegetables of different colors every day?
2	Fruits	BHEHS2: Do you consume fruits of different colors daily?
3	Water	BHEHS3: How often do you drink plain water during the day?
4	Whole grains	BHEHS4: Do you consume whole grains and/or tubers daily?
5	Healthy proteins	BHEHS5: Do you consume animal and/or plant-based proteins daily?
6	Healthy oils	BHEHS6: Do you consume sources of vegetable oils daily, such as olives, avocado, vegetable oil, olive oil, sunflower oil, etc.?

**Note**. For the full instrument, please refer to Appendix A (English version) and Appendix B (Spanish version) of this document.

**Table 3:** Theoretical Foundation of the Bidimensional Structure of the BHEHS-6B.

Dimensions of the BHEHS-6B
**D1: Healthy eating habits related to micronutrients (vegetables, fruits) and water.**
**BHEHS1**: Do you consume vegetables of different colors every day?
**BHEHS2**: Do you consume fruits of different colors daily?
**BHEHS3**: How often do you drink plain water during the day?
**D2: Healthy eating habits related to macronutrients (grains, tubers, proteins, and vegetable fats).**
**BHEHS4**: Do you consume whole grains and/or tubers daily?
**BHEHS5**: Do you consume animal and/or plant-based proteins daily?
**BHEHS6**: Do you consume sources of vegetable oils daily, such as olives, avocado, vegetable oil, olive oil, sunflower oil, among others?

**Note**. D: Dimension.

**Table 4:** Validation based on the content of the brief scale of healthy eating habits (BHEHS-6B).

	Judges (J)							Aiken’s V	Interpretation	
	J1	J2	J3	J4	J5	M	SD	Aiken’s V	VALIDITY	CI (L–U)
**BHEHS1**										
Relevance	3	3	3	3	3	3	0	1.00	VALID	0.70–1.00
Representativeness	3	3	3	3	3	3	0	1.00	VALID	0.70–1.00
Clarity	3	3	3	3	3	3	0	1.00	VALID	0.70–1.00
**BHEHS2**										
Relevance	3	3	3	3	3	3	0	1.00	VALID	0.70–1.00
Representativeness	3	3	3	3	3	3	0	1.00	VALID	0.70–1.00
Clarity	3	3	3	3	3	3	0	1.00	VALID	0.70–1.00
**BHEHS3**										
Relevance	3	3	3	3	3	3	0	1.00	VALID	0.70–1.00
Representativeness	3	3	3	3	3	3	0	1.00	VALID	0.70–1.00
Clarity	3	3	3	3	3	3	0	1.00	VALID	0.70–1.00
**BHEHS4**										
Relevance	3	3	3	3	3	3	0	1.00	VALID	0.70–1.00
Representativeness	3	3	3	3	3	3	0	1.00	VALID	0.70–1.00
Clarity	3	3	3	3	3	3	0	1.00	VALID	0.70–1.00
**BHEHS5**										
Relevance	3	3	3	3	3	3	0	1.00	VALID	0.70–1.00
Representativeness	3	3	3	3	2	2.8	0.45	0.93	VALID	0.62–0.99
Clarity	3	3	3	3	3	3	0	1.00	VALID	0.70–1.00
**BHEHS6**										
Relevance	3	3	2	3	3	2.80	0.45	0.93	VALID	0.62–0.99
Representativeness	3	3	3	3	3	3	0	1.00	VALID	0.70–1.00
Clarity	3	3	2	3	3	2.80	0.45	0.93	VALID	0.62–0.99

**Note**. M = mean; SD = standard deviation; CI = confidence interval, L = lower, U = upper.

**Table 5:** Descriptive statistics and reliability of the Brief Scale of Healthy Eating Habits (BHEHS-6B).

	M	SD	A	K	α	ω
**Healthy Eating Habits** (BHEHS-6B)	22.686	3.283	0.279	−0.531	0.769	0.780
**Dimension 1**	10.807	1.927	0.151	−0.561	0.712	0.724
**Dimension 2**	11.879	1.903	0.005	−0.716	0.703	0.740

**Note.** M = mean; SD = standard deviation; A = asymmetry; K = kurtosis; α = Cronbach’s alpha; ω = McDonald’s omega; dimension 1 = items 1–3; dimension 2 = items 4–6.

**Table 6:** Descriptive analysis of the items and reliability of the items on the brief scale of healthy eating habits BHEHS-6B.

	**M**	**SD**	**A**	**K**	**ω**	**rict**	**Correlation Between Items ^a^**					
							BHEHS1	BHEHS2	BHEHS3	BHEHS4	BHEHS5	BHEHS6
BHEHS1	3.70	0.763	0.210	−0.679	0.720	0.570	-	0.652	0.446	0.516	0.415	0.335
BHEHS2	3.56	0.814	0.273	−0.574	0.733	0.518		-	0.477	0.388	0.338	0.288
BHEHS3	3.56	0.841	−0.109	−0.552	0.747	0.465			-	0.380	0.235	0.378
BHEHS4	4.12	0.690	−0.163	−0.891	0.717	0.598				-	0.725	0.513
BHEHS5	4.17	0.696	−0.244	−0.922	0.738	0.505					-	0.483
BHEHS6	3.59	0.982	−0.391	−0.171	0.753	0.468						-

**Note.** M = mean; SD = standard deviation; A = skewness; K = kurtosis; ritc = corrected item-total correlation; ω = McDonald’s omega (if item is deleted); ^a^ = polychoric correlation.

**Table 7:** Bifactor analysis and hierarchical omega.

CFI	TLI	SRMR	RMSEA	ωh
0.987	0.937	0.025	0.081	0.638

**Note**. CFI = comparative fit index; TLI = Tucker–Lewis index; SRMR = standardized root mean square residual; RMSEA = root mean square error of approximation; ωh = hierarchical omega.

**Table 8:** Confirmatory factor analysis (factor loadings).

Dimension	Items	Factorial Loads	SE	95% CI		Z	*p*	Standardized Factorial Loads
				Lower	Upper			
1	BHEHS1	0.592	0.0522	0.490	0.695	11.34	< 0.001	0.778
	BHEHS2	0.588	0.0557	0.479	0.697	10.56	< 0.001	0.724
	BHEHS3	0.449	0.0601	0.332	0.567	7.48	< 0.001	0.535
2	BHEHS4	0.564	0.0461	0.474	0.655	12.23	< 0.001	0.820
	BHEHS5	0.498	0.0467	0.407	0.589	10.67	< 0.001	0.717
	BHEHS6	0.532	0.0690	0.397	0.668	7.72	< 0.001	0.544

**Note.** SE = standard error; Z = Z-score.

**Table 9:** Confirmatory factor analysis (covariance of the factors).

		Estimator	SE	95% CI		Z	*p*	Standard Estimator
				Lower	Upper			
D 1	D 1	1.000 ᵃ						
	D 2	0.622	0.0646	0.495	0.748	9.63	< 0.001	0.622
D 2	D 2	1.000 ᵃ						

**Note:** D 1 = dimension 1; D 2 = dimension 2; ^a^ fixed parameter.

**Table 10:** Confirmatory factor analysis (model adjustment).

CFI	TLI	SRMR	RMSEA	90% CI for RMSEA	
				Lower	Upper
0.981	0.965	0.0354	0.0601	0.000	0.109

**Note.** CFI = comparative fit index; TLI = Tucker–Lewis index; SRMR = standardized root mean square residual; RMSEA = root mean square error of approximation; test of exact fit: χ^2^(8) = 14.4, *p* = 0.071.

## Data Availability

The datasets generated during and/or analyzed in this study are not publicly available but can be obtained upon request from the corresponding author.

## Abbreviations

The following abbreviations are used in this manuscript:

α	Cronbach’s alpha
BHEHS-6B	Brief Healthy Eating Habits Scale (BHEHS-6B, Version 1.0)
CFI	Comparative fit index
CI	Confidence interval
CNCDs	Chronic non-communicable diseases
EHQ	Eating Habits Questionnaire
HEI	Healthy Eating Index
ω	McDonald’s omega
ωh	Hierarchical omega
ritc	Corrected item-total correlation
RMSEA	Root mean square error of approximation
SD	Standard deviation
SE	Standard error
SRMR	Standardized root mean square residual
TLI	Tucker–Lewis index
WHO	World Health Organization

## Appendix A

**Brief Healthy Eating Habits Scale (BHEHS-6B, Version 1.0)**
**—English Version**

**Instructions:** Below is a list of behaviors related to healthy eating. Please indicate how often you perform each of the actions by placing an X or a check (✓) over the word that best represents your response (*Never, Rarely, Sometimes, Almost Always,* or *Always*). Please remember to select only one option per item.

**Items**			**Never**	**Almost Never**	**Sometimes**	**Almost Always**	**Always**
**1**	**Do you consume vegetables of different colors every day?**		Never	Almost never	Sometimes	Almost always	Always
**2**	**Do you consume fruits of different colors daily?**		Never	Almost never	Sometimes	Almost always	Always
**3**	**How often do you drink plain water during the day?** - *Note*: 1 glass = 250 mL = ¼ liter		Never	1 to 2 glasses of water	3 to 4 glasses of water	5 to 6 glasses of water	7 to 8 glasses of water, or more
**4**	**Do you consume whole grains and/or tubers daily?** *- Examples of **Whole Grains**: whole grain bread, pasta, or rice; oats, quinoa, etc.* - *Examples of **Tubers**: potato, cassava, sweet potato, etc.*		Never	Almost never	Sometimes	Almost always	Always
**5**	**Do you consume animal and/or plant-based proteins daily?** - *Examples of **Animal Protein**: fish, chicken, meat, eggs, dairy products, etc.* - *Examples of **Plant Protein**: legumes, beans, seeds, nuts, etc.*		Never	Almost never	Sometimes	Almost always	Always
**6**	**Do you consume sources of vegetable oils daily, such as olives, avocado, vegetable oil, olive oil, sunflower oil, etc.?**		Never	Almost never	Sometimes	Almost always	Always
		**Copyright Notice.** The BHEHS-6B (Version 1.0) is the intellectual property of the primary author of this study (© 2025, David Javier-Aliaga [D.J.-A.]. All rights reserved). Any use of this scale in scientific publications, research projects, or other forms of dissemination requires prior written authorization (davidjavieraliaga@gmail.com). D.J.-A. is the legal rights holder and the beneficiary of the corresponding royalties.					

## Appendix B

**Escala breve de hábitos de alimentación saludable**
**(EBHAS-6B, versión 1.0)—Versión en español**

**Instrucciones:** A continuación, se presenta una lista de comportamientos relacionados con la alimentación saludable. Por favor, indique con qué frecuencia realiza cada una de las acciones, colocando una aspa (X) o un check (✓) sobre la palabra que mejor represente su respuesta (*Nunca, Rara vez, A veces, Casi siempre o Siempre*). Recuerde seleccionar solo una opción por ítem.

**Ítems**			**Nunca**	**Casi Nunca**	**Algunas Veces**	**Casi Siempre**	**Siempre**
**1**	**¿Consumes diariamente vegetales (o verduras) de diferentes colores?**		Nunca	Casi nunca	Algunas veces	Casi siempre	Siempre
**2**	**¿Consumes diariamente frutas de diferentes colores?**		Nunca	Casi nunca	Algunas veces	Casi siempre	Siempre
**3**	**¿Con qué frecuencia consumes agua pura durante el día?** - ***Nota**. 1 vaso = 250 ml = ¼ de Litro.*		Nunca	1 a 2 vasos de agua	3 a 4 vasos de agua	5 a 6 vasos de agua	7 a 8 vasos de agua, o más
**4**	**¿Consumes diariamente cereales integrales y/o tubérculos?** *- Ejemplos de **Cereales Integrales**:* pan, pasta o arroz integral; avena, quinua, etc. *- Ejemplos de **Tubérculos**:* papa, yuca, camote, etc.		Nunca	Casi nunca	Algunas veces	Casi siempre	Siempre
**5**	**¿Consumes diariamente proteínas de origen animal y/o vegetal?** *- Ejemplos de **Proteína Animal**:* pescado, pollo, carne, huevos, productos lácteos, etc. *- Ejemplos de **Proteína Vegetal**:* granos, legumbres, menestras, semillas, frutos secos, etc.		Nunca	Casi nunca	Algunas veces	Casi siempre	Siempre
**6**	**¿Consumes diariamente fuentes de aceites vegetales, como aceitunas, palta, aceite vegetal, aceite de oliva, aceite de girasol, etc.?**		Nunca	Casi nunca	Algunas veces	Casi siempre	Siempre
		**Nota de derechos de autor.** La BHEHS-6B (versión 1.0) es propiedad intelectual del autor principal de este estudio (© 2025, David Javier-Aliaga [D.J.-A.]. Todos los derechos reservados). Cualquier uso de esta escala en publicaciones científicas, proyectos de investigación u otras formas de difusión requiere autorización escrita previa (davidjavieraliaga@gmail.com). D.J.-A. es el titular legal de los derechos y beneficiario de las regalías correspondientes.					
